# Early non-excisional debridement of paediatric burns under general anaesthesia reduces time to re-epithelialisation and risk of skin graft

**DOI:** 10.1038/s41598-021-03141-x

**Published:** 2021-12-09

**Authors:** Bronwyn Griffin, Anjana Bairagi, Lee Jones, Zoe Dettrick, Maleea Holbert, Roy Kimble

**Affiliations:** 1 NHMRC Centre of Research Excellence-Wiser Wound Care, Menzies Health Institute of Queensland, Griffith University Brisbane, N48 Nathan Campus, Brisbane, QLD Australia; 2 Pegg Leditschke Children’s Burns Centre, Centre for Children’s Burns and Trauma Research, Centre for Children’s Health Research, Brisbane, QLD Australia; 3 Faculty of Health, Queensland University of Technology, Brisbane, QLD Australia

**Keywords:** Paediatric research, Trauma, Health care

## Abstract

Reported advantages of early excision for larger burn injuries include reduced morbidity, mortality, and hospital length of stay for adult burn patients. However, a paucity of evidence supports the best option for paediatric burns and the advantages of non-excisional (mechanical) debridement. Procedural sedation and analgesia in the emergency department is a popular alternative to debridement in operating theatres under general anaesthesia. This study aims to evaluate the association between early (< 24 h post-injury) non-excisional debridement under general anaesthesia with burn wound re-epithelialisation time and skin graft requirements. Cohort study of children younger than 17 years who presented with burns of five percent total body surface area or greater. Data from January 2013 to December 2019 were extracted from a prospectively collected state-wide paediatric burns’ registry. Time to re-epithelialisation was tested using survival analysis, and binary logistic regression for odds of skin graft requirementto analyse effects of early non-excisional debridement in the operating theatre. Overall, 292 children met eligibility (males 55.5%). Early non-excisional debridement under general anaesthesia in the operating theatre, significantly reduced the time to re-epithelialisation (14 days versus 21 days, *p* = 0.029)) and the odds of requiring a skin graft in comparison to paediatric patients debrided in the emergency department under Ketamine sedation (OR: 6.97 (2.14–22.67), *p* < 0.001. This study is the first to demonstrate that early non-excisional debridement under general anaesthesia in the operating theatre significantly reduces wound re-epithelialisation time and subsequent need for a skin graft in paediatric burn patients. Analysis suggests that ketamine procedural sedation and analgesia in the emergency department used for burn wound debridement is not an effective substitute for debridement in the operating theatre.

## Introduction

Significant advances in the survival of paediatric patients with medium to large burn wounds (> 5% total body surface area (TBSA)), were made in the 20th century^1–3^. Sepsis, skin grafting requirements, wound re-epithelialization time have become key clinical outcomes to improve burn wound care beyond survival in developed countries. Following a thermal injury, the compromised barrier function of the skin combined with the anatomical characteristics of paediatric skin, render children more susceptible to inflammation and infection ^4^. The aim of debridement is to remove all non-viable tissue and debris from the injured cutaneous surface. Traditional surgical debridement, using sharp excision, aimed to improve survival by avoiding sepsis, however the sacrifice with this method is the unintentional removal of healthy tissue along with the intentional removal of dead tissue. Non-excisional debridement methods include mechanical (e.g. hydro surgery or abrasion technique) and more recently, enzymatic debridement^5,6^. Mechanical debridement requires an aggressive scrub using gauze, non-cytotoxic cleanser, and water. Once completed, a more accurate assessment of the size and depth of the burn wound is possible, critical considerations of burn management^7^.

Many studies have demonstrated that, delayed burn wound re-epithelialisation is associated with an increased risk for hypertrophic scar formation in children^8–10^. Multiple factors have been found to influence this critical time to re-epithelialisation time in children. Hence, clinicians consider the timing, setting and analgesia at initial debridement of medium to large burn wounds to optimise outcomes such as re-epithelialisation time and requirement for skin graft. Time to wound debridement is dependent on the consideration of a multitude of factors including patient stability, injury severity, body location and TBSA^11^. Early debridement is thought to reduce the toxic and bacterial burden from a burn wound^12–14^ and within 24 h of injury has been associated with significantly reduced re-epithelialisation time in adults ^15^. In children, early excision with immediate wound closure was associated with improved survival, shorter hospitalisation^3,13,16^ and found to be safe and effective within 72 h of injury^1^. At the study site, a quaternary paediatric hospital and burns centre, greater than 20,000 paediatric burns patients have been treated over the last 20 years with only two children succumbing to their injuries.

Furthermore, there are several factors in the consideration for early debridement including burn severity, staff expertise, pain, conforming dressing, available resources, location, time of day/week/weekend, and type of analgesia that will be administered. Sub-optimal pain management has been shown to delay paediatric burn wound re-epithelialisation ^17^. In addition, a recent study reported that parental acute psychological distress influences child procedural-related pain distress ^18^. Often, minor (TBSA < 5%), and a proportion of medium to large TBSA burns, are initially managed in the emergency department (ED) with procedural sedation and analgesia (PSA). The level of PSA ranges from minimal (anxiolysis with impaired cognitive function) to moderate, where the child would have reduced level of consciousness, respond to verbal commands and maintain adequate spontaneous ventilation ^19,20^. The resurgence of paediatric PSA with ketamine, either as a single drug or in combination with other PSA agents ^21^ is due to its potent anaesthetic and analgesic properties and low incidence cardiorespiratory adverse effects^22–27^ when compared to opioids. For these reasons, ketamine PSA is a popular choice by clinicians treating paediatric burns wounds in EDs ^21,28–30^. Despite this, ‘emergence reaction’ after ketamine is a well-documented adverse reaction. The rate of hallucination when emerging from a dissociative state occurs at rates between 5 and 14% ^31^, and is reported to be transient and mild in children^32^. However, little is known on the efficacy of longer term protective factors of medical trauma.

This study aims to evaluate the effect of non-excisional, mechanical debridement within 24 h of paediatric burns injuries greater than or equal to 5% TBSA, under general anaesthesia in the operation theatre on wound re-epithelialisation and skin graft requirements.

## Objectives

The objectives of this study were as follows.

1. *Primary* The effect of timing, setting and analgesia for non-excisional debridement of acute, medium to large (≥ 5% TBSA), paediatric burn injuries on time to re-epithelialisation.
2. *Secondary* The effect of timing, setting and analgesia for non-excisional debridement of acute, medium to large (≥ 5% TBSA), paediatric burn injuries on skin graft requirements.

## Methods

A single-centre, retrospective, cohort study using prospectively collected data from the *(De-identified)* Paediatric Burns Registry was conducted at a paediatric burn’s referral centre in *(De-identified)*, Australia. All children who presented to the study site, between January 2013 and December 2019, younger than 17 years, with TBSA ≥ 5%, of any burn mechanism were eligible for inclusion. All eligible families who presented to the burns outpatient department or inpatient ward were approached for consent and data collection. Parents who declined for their child’s data to be entered into the Paediatric Burns Registry were excluded from this study. Registry data collection consists of demographic and injury characteristics as well as clinical interventions, hospital interactions and clinical outcomes. Children with full thickness burns were excluded due to their certainty of requiring a skin graft and conversely superficial burns were excluded due to their unlikely requirements of skin grafting ^33^

All children taken to theatre within 24-h post-burn for an initial, non-excisional debridement in theatre received general anaesthesia. The non-excisional debridement intervention uses an aggressive washing technique with sterile water, soap-free surfactant cleanser (QV Cleanser, Melbourne, Australia) ^34^, and sterile gauze sponge (Ray-Tec, Johnson & Johnson, NJ, USA) for the removal of all non-viable tissue from the burn wound. This was followed by immediate wound closure with an appropriate cover such as a silver impregnated, or biological dressing as determined by the treating surgeon. Children were then subsequently managed as either in-patient or outpatients, dependent on the severity of burn injury or other concern. The treating burns surgeon determined when burn wound achieved spontaneous re-epithelialisation ≥ 95% or requirement for a skin graft.

Reporting of this cohort study conforms to the Strengthening the Reporting of Observational Studies in Epidemiology (STROBE) Statement ^35^. Once the study was approved by the Children’s Health Queensland Hospital and Health Service Human Research Ethics Committee (HREC/16/QRCH/61. SSA/16/QRCH/61), data were extracted and deidentified prior to analysis. The study was conducted in accordance to the principles of the Declaration of Helsinki. Written informed consent was obtained from the legal guardians at the time of data registry inclusion.

### Data management

Data for each patient was extracted from the *(De-identified)* Paediatric Burns Registry and included socio-demographic data, TBSA%, burn depth, age of burn in hours at debridement, initial dressing applied, analgesia at debridement, setting of initial debridement, time to re-epithelialisation in days and incidents of skin graft requirements. Data collection was from the time of first presentation to a health service, up until wound re-epithelialisation was achieved or skin grafting undertaken. Data was captured with and stored in FileMaker (Claris International Inc., NSW, Australia).

### Statistical methods

The timing, location and analgesia at initial wound debridement were selected as variables of interest for this investigation, with key outcomes being differences in time to re-epithelialisation and need for skin grafting. The dataset was divided into three groups: ‘OT < 24 h’, ‘ED Ketamine PSA’, and ‘Other Settings’ (comparator). The ‘OT < 24 h’ group included paediatric burn patients taken to OT for non-excisional wound debridement under general anaesthetic within the first 24-h following burn injury. The ‘ED Ketamine PSA’ group included children whose debridement were completed in the ED with Ketamine PSA. The ‘Other settings’ group included all other debridement that were not completed in the operation theatre under general anaesthesia within 24 h of injury or in the ED under Ketamine PSA.

Descriptive analysis was carried out for all key variables. Chi-square tests were performed to examine the relationships between purely categoric variables and the context of debridement (OT24hrs, ED Ketamine PSA or Other). Due to non-normal distributions, Kruskal–Wallis tests were used for continuous variables.

Binary logistic regression was used to determine the associations between the three treatment groups and requirement for skin graft, after controlling for burn severity as indicated by TBSA and burn depth.

Cox proportional hazards model was used to examine the effect of treatment group on wound re-epithelialisation time, adjusted for burn severity among those who did not receive a skin graft. A sensitivity analysis was performed to examine the impact of inclusion of patients with a skin graft on the findings of time to re-epithelialisation to include, as skin grafts are an augmented wound closure. Following consultation with the study centre burn surgeons, a dummy value of 28 days was selected to estimate an average time to re-epithelialisation in order to account for all large burns in the cohort. Sensitivity analysis (Supplementary File 1) indicated no substantial changes in the conclusions and the initial model was retained. A value of p < 0.05 was considered statistically significant. Data was analysed with SPSS 27 (IBM Corporation, Armonk, NY, USA) software.

### Ethics approval and consent to participate

The study was approved by the Children’s Health Queensland Hospital and Health Service Human Research Ethics Committee (HREC/16/QRCH/61).

### Consent for publication

Consent for inclusion in the database was obtained at data collection. Patient information was deidentified while undergoing statistical analysis, maintaining patient privacy and confidentiality.

## Results

### Participants

Two hundred and ninety-two paediatric burn patients met the inclusion criteria for the study (i.e., aged ≤ 17 years with a burn TBSA ≥ 5%) and were extracted from the *(De-identified)* Paediatric Burns Registry. Demographic details of the sample population are presented in Table 1. Children under the age of four accounted for over 68% of participants included in this investigation. Males were slightly overrepresented in the sample population—accounting for more than 54% of children overall. No significant differences were found between the ED Ketamine PSA, Other Setting, or the OT < 24hrs for first aid, gender, age, or time to debridement.

**Table 1:** Characteristics of patients: debrided in the ED under ketamine PSA, debrided in other settings, and debrided in the OT within 24 h.

	Debridement in the ED with ketamine *n* = 28 N (%)	Debridement other settings *n* = 220 N %	Debridement in OT within 24 h *n* = 44 N %	*p* value
**Burn depth**				
DDPT	16 (57.1)	107 (48.6)	33 (75)	
SPT	12 (42.9)	113 (51.4)	11 (25)	0.005
TBSA % Median (IQR)	9.5 (8–12.8)	6 (5–9)	11.5 (7.3–16)	< 0.001
**Gender**				0.665
Male	16 (57.1)	125 (56.8)	21 (47.7)	
Female	12 (42.9)	92 (41.8)	21 (47.7)	
Missing	–	3 (1.4)	2 (4.5)	
**First aid**				0.944
Yes	21 (75)	168 (76.4)	32 (72.7)	
No	6 (21.4)	52 (23.6)	11 (25)	
Missing	1 (3.6)	–	1 (2.3)	
**Age (years)**				0.081
0–4	23 (82.1)	143 (65)	34 (77.3)	
5–10	4 (14.3)	46 (20.9)	3 (6.8)	
> 10	1 (3.6)	31 (14.1)	7 (15.9)	
Time to re-epithelialisation (Days) Median (IQR)	21 (12–34)	17 (12–23)	14 (10–19)	0.020
Time to debridement (h:min)	5:07 (3:26–11:27)	5:29 (3:44–9:27)	9:44 (2:57–22:35)	0.730
**Definitive dressing applied**				
Acticoat	13 (46.6)	125 (56.8)	29 (65.9)	
Mepilex Ag	10 (35.7)	72 (32.7)	3 (6.8)	
Combined silver	5 (17.9)	18 (8.2)	1 (2.3)	
Biobrane	–	–	8 (18.2)	
RECELL	–	1 (0.5)	2 (4.5)	
Flamazine	–	2 (0.9)	–	
Not recorded	–	2 (0.9)	1 (2.3)	
Grafted	13 (46.4)	37 (16.8)	9 (20.5)	0.001

*DDPT* deep dermal partial thickness, *SPT* superficial partial thickness, *TBSA* total body surface area, *ED* emergency department, *OT* operating theatre, *IQR* interquartile range.

^a^Combined Silver = Acticoat + Mepilex Ag + Hypafix applied to burns.

### Effect of non-excisional debridement on time to re-epithelialisation

A significant difference in time to re-epithelialisation was identified between paediatric patients taken to theatre within 24 h in comparison to those debrided in the ED under Ketamine PSA (p = 0.029). Median time to re-epithelialisation for children taken to theatre for debridement under general anaesthetic was 14 days (IQR 10–19) versus 21 days (IQR: 12–34), *p* = 0.029 for patients debrided in the ED under Ketamine PSA. Median time to re-epithelialisation for children in the Other Settings group was equal to 17 days (IQR: 12–23).

**Figure 1:**
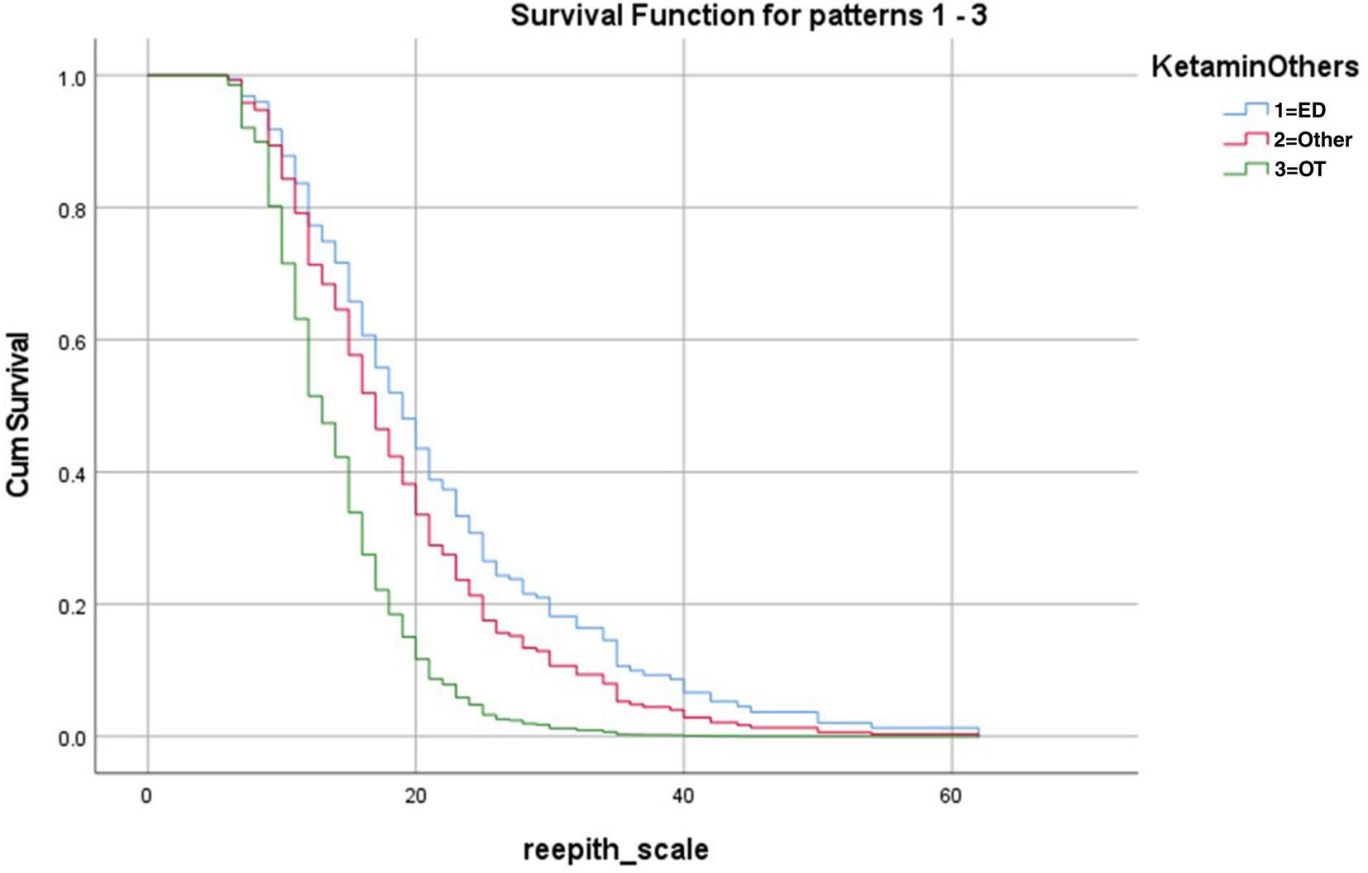
Effect of non-excisional debridement on re-epithelialisation time (Cox survival Plot).

Cox regression analysis determined there was a significant effect of the setting of the initial non-excisional debridement of burn injuries on burn wound time to re-epithelialisation after adjusting for burn severity (Table 2). In comparison to children who received non-excisional wound debridement in the OT within 24-h post-burn, paediatric patients who underwent non-excisional debridement under Ketamine PSA in the ED had a 61% reduced chance of reaching 95% re-epithelialisation (Hazards Ratio = 0.39, 95% CI 0.21–0.72, *p *< 0.001). Furthermore, children debrided in ‘Other Settings’ had a 49% reduced chance of reaching 95% re-epithelialisation in comparison to children who received non-excisional debridement under general anaesthetic in theatre within 24 h (Hazards Ratio = 0.51, 95% CI 0.33–0.78, *p* = 0.001). Deep dermal partial thickness (DDPT) injuries demonstrated a significant effect on time to re-epithelialisation (*p* < 0.001), confirming their significant incorporation into the model. A Cox Regression Survival Plot (Fig. 1) demonstrates the significant shortened time for the OT<24hrs Group compared to Other Settings and Ketamine PSA in ED Groups.

**Table 2:** Time to re-epithelialisation Cox regression (hazard ratios) n = 233.

Variable	Sub-group	Hazard ratio (95% CI)	*p* value
TBSA		0.97 (0.93–1.01)	0.150
Burn depth	DDPT	0.52 (0.39–0.67)	< 0.001
	SPT	1	
Debridement sub-group	Ketamine in the ED	0.39 (0.21–0.72)	< 0.001
	Other	0.51 (0.33–0.78)	0.001
	OT within 24 h	1	

NB: All grafted patients N = 59, were excluded from this analysis.

*DDPT* deep dermal partial thickness, *SPT* superficial partial thickness, *TBSA* total body surface area, *ED* emergency department, *OT* operating theatre, *CI* confidence interval.

*1 = reference group for regression.

The additional model, using a proxy value of 28 days for grafted patients reflected similar patterns of Hazard Ratios described in Table 2 (Supplementary File 1).

### Effect of non-excisional debridement on requirement for skin graft

In the binary regression, children in the Ketamine PSA group had almost seven times the odds of requiring a skin graft compared to those receiving non-excisional debridement under general anaesthesia within 24 h of injury (Odds Ratio = 6.97, 95% CI 2.14–22.67, *p *< 0.001), even after controlling for variables known to influence rates of grafting such deep partial thickness burn depth and TBSA% (Table 3). No significant difference in odds of split thickness skin grafting was identified between paediatric patients in the Other Settings group compared to those in the OT<24hrs group (Odds Ratio = 0.51, 95% CI 0.33–0.78, *p* = 0.126).

**Table 3:** Odds of grafting following non-excisional debridement n = 292.

Variable	Sub-group (N)	Odds ratio (95% CI)	*p* value
TBSA		1.14 (1.05–1.24)	0.001
Burn depth	DDPT	6.6 (3.03–14.18)	< 0.001
	SPT	*1	
Debridement sub-group	Ketamine in the ED	6.97 (2.14–22.67)	< 0.001
	Other	2.1 (0.81–5.62)	0.126
	OT within 24 h	*1	

*DDPT* deep dermal partial thickness, *SPT* superficial partial thickness, *TBSA* total body surface area, *ED* emergency department, *OT* operating theatre, *CI* confidence interval.

*1 = reference group for regression.

## Discussion

This study is the first to demonstrate that early initial, non-excisional debridement of acute paediatric burns under general anaesthesia in an operating theatre, significantly reduces the wound re-epithelialisation time and subsequent requirements for skin graft. Non-excisional burn wound debridement completed in the operating theatre within 24 h of burn injury resulted in a wound re-epithelialisation time of 7 days faster when compared to Ketamine PSA in the ED. The odds for requiring a skin graft were significantly increased when non-excisional debridement was not completed in theatre under general anaesthetic within 24 h of injury. These findings add to the evidence supporting early debridement of acute burn injuries in children ^3,12,37,38^.

The evolution and improvements of paediatric burn care have been reflected by an exceptional increase in survival rates over the last 50 years. Thus, expanding the focus of burn care to decreasing the risk of scar formation. Chipp et al. demonstrated the linear relationship between time to re-epithelialisation and risk of scaring with every additional day taken to re-epithelialise, multiplying the risk of hypertrophic scaring by 1.138^10^. In addition to this Chipp et al. challenged traditional dogma of healing within 3 weeks to be oversimplified in the paediatric cohort, emphasizing that every effort should be made to reach re-epithelialisation as quickly as possible^10^.

The initial phase of burn wound healing is typified by inflammation and haemostasis that confine the extent of injury and cleanse the wound ^39^. Burn wound conversion causes deepening of the burn wound due to ongoing ischaemia and inflammation ^40^. Early tangential excision is thought to address this inflammatory phase by removal of non-viable tissue from the wound, first described by Janzekovic ^41^. Lu et al. demonstrated the influence of tangential excision within 24 h post-burn injury of deep partial wounds was a significant reduction of inflammatory markers (IL8, MPO and MDA) when compared to non-debrided areas of the wound ^14^. We hypothesise that non-excisional debridement, in comparison to excisional debridement, is likely to preserve more healthy tissue and contribute towards the removal of the considerable burden of these inflammatory markers.

In the ED, wound debridement may occur with the parent present during the procedure. Some parents experience distress observing this process. There is evidence to support that parental distress and anxiety directly correlates to the child’s burn wound healing time^42,43^. It is postulated that general anaesthesia in the operation theatre provides an environment for complete burn wound debridement, adequate wound closure, and optimal peri-procedural analgesia. Traditionally, clinicians have been reluctant to subject young children to frequent general anaesthesia due to concerns for neurotoxicity after exposure to anaesthetic drugs^44,45^. Recently, three large paediatric studies (GAS^46^, PANDA^47^, and MASK ^48^) have identified no correlation between single anaesthesia exposure and reduced cognition^45^. Whilst this is a promising finding for the safety of children, limited research has been conducted examining the influence of debridement setting and analgesia within 24 h of injury on clinical outcomes such as re-epithelialisation time and skin graft requirements for medium to large burns.

Debridement under general anaesthesia provides a controlled environment where peri-procedural analgesia can be optimised. Brown et al. showed that wound re-epithelialisation was delayed by 2.2% for every increase of one point on the Faces Pain Scale Revised ^17^. It is postulated that whilst under a general anaesthesia, the injured child is not able to formulate a memory of the painful procedure that may contribute towards increased anticipatory distress ^49^ during subsequent dressing changes. Further studies would be required to explore this hypothesis. Another proposed benefit of general anaesthesia for initial debridement is that burn surgeons can select the most appropriate wound management approach and achieve complete coverage of the burn wound. This is not always possible in a busy emergency department, for a child who has been given peri-procedural analgesia with or without adjunct distraction techniques.

Efforts to address the complex physiological activity of an acute burn injury, specifically to disrupt wound progression, are increasingly visible in scientific burns literature. The early application of negative pressure wound therapy in paediatric burn wounds has shown decreased time to re-epithelialisation, with suggested cost savings due to decreased proportions of skin grating requirements ^36^. Additionally, effective adherence to 20 min of cool running water within the first three hours of burn injury has resulted in significantly reduced odds of skin grafting amongst other patient outcomes ^50,51^. More recently Holbert et al. highlighted the characteristics of burn wounds associated with higher pain levels ^52^. Acknowledging the impact risk factors and interventions have on the time to re-epithelialisation and subsequent risk of scarring are important considerations in tailoring acute burn treatment pathways. Bundling these individual interventions together may lead to additional improvements in patient outcomes. More studies would be necessary to explore this hypothesis.

The setting for this study is the sole paediatric tertiary burns centre, housing five burns surgeons, treating > 1200 new burns per annum, covering a land mass of 1.85 million km^2^/715,447.3 miles^2^. This study was performed to enable decision makers with evidence to facilitate access to the best treatment options for optimal clinical outcomes and define treatment pathways into the future. In patients who went to operating theatre in less than 24 h, there were two patients included who received RECELL. Although a recent literature review could not reach a definitive role of autologous skin cell suspension in re-epithelialisation, there is widespread anecdotal acknowledgment of the positive experience of the intervention. However, with only two participants receiving RECELL, it is highly unlikely to have impacted the reliability of results presented.

The cost and access to an operating theatre is not always an easy accomplishment in a busy tertiary hospital. Whilst this intervention can be perceived as an early burden on hospital resources, other studies have shown that early intervention investments improve the longer term patient outcome benefits and ultimately overall cost effectiveness ^53,54^. To better define this benefit, future work should incorporate a formalised cost effectiveness analysis, to strengthen discussions with hospital executives to consider prioritising operating theatres for this intervention.

There are noteworthy limitations in this study. Firstly, the observational data set is at risk of selection bias associated with restricting the data selection and subsequent analysis to participants with completed data for outcomes. Secondly, although detailed training was provided for data collectors, the possibility of variability in data entry into the proformas cannot be eliminated. Lastly, the cohort is small and results could be bolstered with a greater sample size in subsequent studies in this area.

## Conclusion

Early non-excisional debridement of acute burns under general anaesthesia in children reduces wound re-epithelialisation time and requirements for skin grafting. Effective non-excisional debridement can be achieved under general anaesthesia, aggressive mechanical debridement with warm water, sterile surgical gauze, and a soap-free surfactant cleanser.

## Supplementary Information

Supplementary Information.

## Abbreviations

TBSA: Total body surface area
CI: Confidence interval
IQR: Inter-quartile range
OR: Odds ratio
FT: Full thickness
DDPT: Deep dermal partial thickness
SPT: Superficial partial thickness
OT: Operating theatre
IQR: Interquartile range
ED: Emergency Department
GA: General anaesthesia
PSA: Procedural sedation and analgesia
M: Male
F: Female
IL8: Interleukin-8
MPO: Myeloperoxidase
MDA: Malondialdehyde
GAS: General Anaesthesia Spinal Trial (46)
PANDA: Paediatric Anaesthesia Neurodevelopmental Assessment Trial (47)
MASK: Mayo Anaesthesia Safety in Kids Trial (48)
