# Supplementary material for: Early non-excisional debridement of paediatric burns under general anaesthesia reduces time to re-epithelialisation and risk of skin graft
Source: Sci Rep. 2021 Dec 9;11:23753. doi: 10.1038/s41598-021-03141-x (PMC8660833; doi:10.1038/s41598-021-03141-x)
Supplement: Supplementary file 1 — Supplementary Information. [file 41598_2021_3141_MOESM1_ESM.docx]

**Supplementary File 1. Time to re-epithelialisation Cox regression (hazard ratios)**

| Variable | Sub-group | Hazard Ratio (95% CI) | *p* value |
| --- | --- | --- | --- |
| TBSA | N = 292 | 0.9 (0.89 – 0.97) | 0.001 |
| Burn Depth | DDPT (*n* = 156)  SPT (*n* = 136) | 0.4 (0.31 – 0.53)  1 | <0.001 |
| Debridement Sub-group | Ketamine in the ED (*n* = 28)  Other (*n* = 220)  OT within 24 Hours (*n* = 44) | 0.3 (0.16 – 0.56)  0.47 (0.31 – 0.73)  1 | <0.001  0.001 |

**A proxy time to re-epithelialisation of 28 days was use for grafted patients in this analysis**.
